# Clinical characteristic of myelin oligodendrocyte glycoprotein antibody associated cortical encephalitis in adults and outcomes following glucocorticoid therapy

**DOI:** 10.3389/fnagi.2022.1076361

**Published:** 2023-01-04

**Authors:** Yuqing Wu, Hao Zhou, Xiaojiao Ci, Liuyu Lin, Da Zhang, Jie Lu

**Affiliations:** Department of Neurology, The Affiliated Brain Hospital of Nanjing Medical University, Nanjing, China

**Keywords:** MOG, cortical encephalitis, clinical feature, MRI, glucocorticoids therapy

## Abstract

**Objective:**

To describe the clinical and radiological features, as well as outcomes following glucocorticoid therapy and recurrence in adults suffering from cortical encephalitis associated with myelin oligodendrocyte glycoprotein (MOG) antibody.

**Methods:**

The clinical information of nine adult patients suffering from cortical encephalitis associated with MOG antibody admitted to the Affiliated Brain Hospital of Nanjing Medical University from 2020 to 2022 was systematically reviewed. The clinical symptoms, laboratory data, imaging results, outcomes following glucocorticoid therapy and recurrence were evaluated.

**Result:**

A total of 9 patients positive for MOG antibody and suffering from cortical encephalitis were included in our study (55.6% men, median age 29 years, 15–57 years). The most common clinical symptoms included headache (77.8%), fever (66.7%), and generalized seizures (55.6%). Some patients also experienced limb shaking (22.2%), leg numbness (22.2%), transient motor aphasia (11.1%), and vision loss (11.1%). The main features of cerebrospinal fluid () examination were increased intracranial pressure, pleocytosis, and elevated cerebrospinal fluid (CSF) protein. In addition, N-methyl-D-aspartate receptor (NMDAR) and MOG antibodies were found in the CSF of 3 patients, and NMDAR, MOG, and glial fibrillary acidic protein antibodies were found in the CSF of 1 patient. All patients were subjected to magnetic resonance imaging (MRI) and the images of eight of them showed T2 and/flair image hyperintense lesions, three showed meningeal or lesion enhancement and four showed white matter lesions, which were mostly located in the midline structures (75%). All patients received glucocorticoid therapy in the acute phase and in remission, and eight of them received an intravenous high dose of methylprednisolone, including one patient who received a simultaneous immunoglobulin therapy. One patient was treated with low-dose prednisolone tablets. Seven (77.8%) patients were wholly recovered at discharge, and 2 (22.2%) patients were left with slight symptoms. During the median 9-month follow-up (range: 2–36 months), 2 (22.2%) patients developed recurrence.

**Conclusion:**

The clinical manifestations of adult MOG antibody-associated cortical encephalitis were significantly different from those of the typical MOG antibody-associated disease (MOGAD). Patients in the acute phase of the disease were prone to show signs similar to central nervous system infection, requiring clinicians to have the ability to recognize the disease to avoid misdiagnosis. In addition, seizures were common in MOG antibody-related encephalitis, and the type of seizures was age-related. Brain MRI results showed that the distribution of cerebral cortex lesions was closely related to the classification of cortical encephalitis. Based on the patient’s response to the treatment, glucocorticoid therapy was effective against MOG antibody-associated cortical encephalitis, which is consistent with the treatment response and clinical prognosis of MOGAD. Therefore, our opinion was that MOG antibody might be the “responsible antibody” in MOG antibody-associated cortical encephalitis, although further studies are needed to confirm this hypothesis.

## Introduction

Myelin oligodendrocyte glycoprotein (MOG) is expressed in the outermost layer of the myelin sheath, and is involved in the organization and maintenance of the myelin sheath of neurons (Androutsou et al., 2018), but it is still controversial whether the presence of the MOG antibody causes an autoimmune demyelinating disease of the central nervous system (CNS; Marignier et al., 2021). However, MOG has been established as an antigenic target in animal models of experimental autoimmune encephalomyelitis, and relevant studies proposed that MOG antibodies are involved in the pathogenesis of inflammatory demyelinating disease as revealed by animal models (Merkler et al., 2006; Reindl et al., 2013; Ramanathan et al., 2016; Stassart et al., 2016). Since the advent of highly specific cell-based assays, MOG antibodies have frequently been detected in patients with optic neuritis, neuromyelitis optica, myelitis, acute disseminated encephalomyelitis (ADEM), brainstem encephalitis, and cortical encephalitis. However, it is rarely detected in patients with multiple sclerosis and aquaporin-4 antibody positive neuromyelitis optica spectrum disorders (Kitley et al., 2012; Reindl et al., 2013; Sato et al., 2014; Fujimori et al., 2019, 2020a). These pieces of evidence suggest that MOG antibodies define a novel demyelinating disease of the CNS that has been described as MOG antibody associated disease (MOGAD).

MOGAD tends to occur in children, and it is mostly manifested as ADEM. MOG antibody-associated cortical encephalitis was firstly reported by Fujimori et al. (2017), and subsequently, similar cases were gradually reported (Adachi et al., 2018; Ikeda et al., 2018; Budhram et al., 2019); then MOG antibody-associated cortical encephalitis was proposed. This broadens the clinical scope of MOGAD. Different from classical ADEM, MOG antibody-associated encephalitis is well-circumscribed and cortical-based in neuroimaging, so it is called cortical encephalitis or MOG antibody-associated autoimmune encephalitis (AE) by relevant researchers (Yao et al., 2022). However, some patients do not show any abnormality in imaging manifestations (Yao et al., 2022). Although few studies reported the presence of MOG antibody-associated cortical encephalitis in some patients, the knowledge of the phenotype is still extremely limited. Therefore, the objective of this study was to describe the clinical and radiographic features of MOG antibody-associated cortical encephalitis in adults, the outcomes following glucocorticoid therapy, and the recurrence during the follow-up period.

## Materials and methods

### Patients

This was a retrospective study involving adult patients with MOG antibody-associated cortical encephalitis who were admitted to the Brain Hospital Affiliated to Nanjing Medical University from 2020 to 2022. Patients were included if they met the following inclusion criteria: (a) age ≥ 15 years; (b) diagnosed with AE but not with ADEM; (c) MOG antibodies present in the serum and/or cerebrospinal fluid (CSF) tested by indirect immunofluorescence test (IIFT). The exclusion criteria were the following: (a) patients who also have other MOGAD phenotypes or other demyelinating diseases during the acute episode; (b) lost to follow-up.

The study was approved by the Ethics Committee of the Affiliated Brain Hospital of Nanjing Medical University, and all patients provided a written informed consent to agree to all the procedures in the study.

### Collection of clinical data

The demographic, clinical manifestation, and laboratory data of all patients were collected and analyzed. The presence of CNS demyelinating antibodies [AQP4, MOG, myelin basic protein (MBP) or AQP4, MOG, glial fibrillary acidic protein (GFAP)] and AE antibodies (NMDAR, AMPA1, AMPA2, LG1, CASPER2, GABABR) was evaluated in all patients. Titers in the serum and CSF samples were assessed using IIFTby Omeng Weiyi Medical Laboratory (Hangzhou, China). Serum antibody titer ≥1:10 was defined as positive, and CSF antibody titer ≥1:1 was defined as positive. Routine, biochemical, immunological, and cytological tests of CSF were performed in all patients.

All patients were evaluated for infectivity. Routine detection of serum infectious disease combination and TORCH combination were performed in patients， and the presence of bacteria in the CSF was routinely evaluated by Gram staining, acid fast bacilli stain and ink stain. CSF bacterial culture, fungal culture, mycobacterial culture, and cryptococcal latex agglutination test were performed in patients with CSF pleocytosis, and if necessary, next generation sequencing was also performed to detect infectious pathogens. None of them had pathogen infections.

All patients received glucocorticoid therapy in the acute phase and in remission, and the effect of glucocorticoids in the acute phase was assessed using the Modified Rankin Scale (mRS) score at discharge. The treatment was performed by anintravenous injection of methylprednisolone 500 mg for 5 days from the initial diagnosis. The dosage reduction plan of oral steroids in remission started with oral prednisone 60 mg, then it was decreased to one tablet every 2 weeks up to 30 mg, and then decreased to one tablet every week. A change in mRS score of at least 1 point was considered as effective. Relapse was defined as the appearance of any new CNS symptom or sign lasting at least 24 h and supported by clinical laboratory or imaging findings.

Brain magnetic resonance imaging (MRI) was performed by a 3.0 T scanner in all patients. Gadolinium was intravenously injected at a rate of 0.1 mmol/kg and MRI was performed immediately after the administration. The T1 weighted, T2 weighted, T2 fluid-attenuated inversion recovery (Flair) sequence, enhanced images and diffusion weighted (DWI) sequence of brain MRI were analyzed.

## Result

### Demographics and clinical features

Nine patients with MOG antibody-positive associated cortical encephalitis were identified. The median age at the onset was 29 years (range, 15–57 years), and the patients were 4 females (44.4%) and 5 males (55.6%). Demographic and clinical data are summarized in Table 1.

**Table 1:** Clinical features of nine patients with cortical encephalitis positive for the MOG antibody.

Pat	Age/ sex	Clinical symptoms of acute phase				Laboratory finding							MRI finding					Treatment	mRS	Replase/(m)
		Headahe	Fever (37.3–39°C)	Seizure	Other symptoms	CSF				Mog ab		Other abs	Cortical lesion	Lesion pattern	GM	WM	Enhancement			
						pressure	WC	protein	OB	serum	CSF									
1	21/M	+	+	Generalized seizure	Palsy of two legs, motor aphasia	270	285	0.61	NA	1:32	1: 3.2	−	Frontal and parietal	Bilateral	−	Juxta cortical WM	+	HIMP	1	0/24
2	15/M	−	−	Generalized seizure	−	70	12	0.64	NA	1:1000	−	−	−	−	bilateral thalamus	−	+	HIMP	0	0/36
3	29/M	+	+	Generalized seizure	−	NA	112	0.45	−	−	1:100	NMDAR(CSF:1:10)	Frontal	Bilateral	−	Anterior commissure gray cinereum	NA	HIMP	0	1/26
												GFAP(CSF:1:32)								
4	29/M	+	−	Focal with secondary generalized seizures	−	212	156	0.64	+	1:32	1:32	NMDAR(CSF:1:10)	−	−	−	Central semiovale and corona radiata	NA	HIMP + IVIG	0	0/9
5	33/F	+	+	Generalized seizure	−	80	8	0.38	+	1:10	1:3.2	NMDAR(CSF:1:1)	Right temporal and parietal	Unilateral	−	−	+	HIMP	0	0/4
6	23/F	+	−	−	−	190	132	0.43	+	1:32	−	−	Left frontal, temporal and parietal	Unilateral	−	−	−	HIMP	0	0/7
7	26/F	+	+	−	Vision loss, limb jitter	500	159	0.44	−	1:100	1:1	NMDAR (CSF:1:1)	Left frontal	Unilateral	−	−	−	HIMP	1	0/13
8	35/F	+	+	−	−	140	276	0.61	NA	1:32	NA	−	−	−	−	−	NA	Prednisone	0	1/6
9	57/M	−	+	−	Palsy of two legs, limb jitter	72	55	0.76	NA	1:32	−	−	Right occipital	Unilateral	Bilateral basal ganglia	By the lateral ventricle	−	HIMP	0	0/2

Pat, patient; M, male; F, female; WC, white cell; OB, oligoclonal bands; MOG ab, myelin oligodendrocyte glycoprotein antibody; CSF, cerebrospinal fluid; NMDAR, N-methyl-D-aspartate receptor; GFAP, glial fibrillary acidic protein; GM, gray matter; WM, white matter; HIMP, high-dose intravenous methylprednisolone; IVIG, intravenous immunoglobulins; mRS, modified Rankin scale; NA, not available; MRI, magnetic resonance imaging; abs, antibodies.

The most common symptoms in these patients were headache (7/9, 77.8%), fever (6/9, 66.7%) and seizure (5/9, 55.6%). Generalized seizure (4/5, 80%) was the most common type of seizure, followed by partial seizure followed by generalized seizure (1/5, 20%). Some patients also showed limb jitter (2/9, 22.2%), palsy of the two legs (2/9, 22.2%), transient motor aphasia (1/9, 11.1%) and vision loss (1/9, 11.1%).

### Laboratory features

All patients were positive for MOG antibodies in the serum and CSF during the acute phase, with antibody titers ranging from 1:3.2 to 1:1,000 (Table 1). Antibody titers varied widely among patients and were not found to be associated with disease severity or treatment response. Among the nine patients, three were positive for CSF MOG antibody and NMDAR antibody (Pat4, Pat5, and Pat7), and a patient was also positive for CSF MOG antibody, NMDAR antibody and GFAP antibody (Pat3). The clinical symptoms of these four patients were not unusual compared to the other patients. As regards CSF examination, 3/8 patients (37.5%) had increased intracranial pressure (> 200 mm H_2_O) with a median of 270 mm H_2_O (range, 212–500 mm H_2_O), 8/9 (88.9%) patients had CSF pleocytosis (> 8 white cell count/μl) with a median of 132 cells/μl (range, 12–285 cells/μl), 8/9 patients (88.9%) had increased protein (> 0.4 g/L) with a median of 0.63 g/L (range, 0.43–1.06 g/L), 3/5 patients (60%) were positive for oligoclonal bands in CSF.

### Radiological features

**Figure 1:**
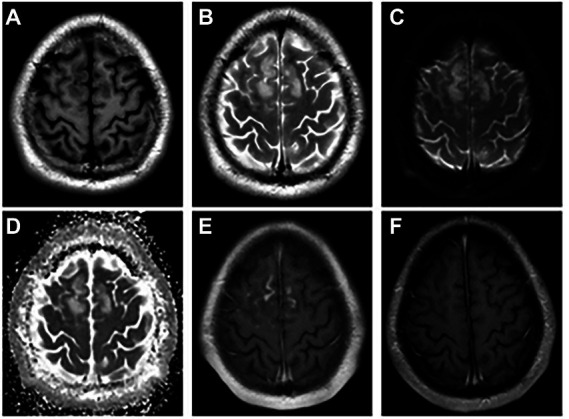
The abnormal MRI was obtained from patient 1. A 21-year-old woman with serum and CSF MOG antibodies, presenting with low-to-moderate fever, seizures, headache, transient motor aphasia, and bilateral palsy of two legs. Bilateral frontal–parietal falciparum cortex are swollen, axis T1-weighted **(A)** and T2-weighted images **(B)** show significant signal alterations in bilateral frontal–parietal cortex and juxta cortical white matter. DWI **(C)** and ADC image show hyperintense signal **(D)**, and enhancement the corresponding of lesions are enhanced **(E)**. Re-examination MRI enhancement **(F)** at discharge shows enhanced lesions disappeared. DWI = Diffusion Weighted Imaging, ADC = apparent diffusion coefficient, MOG = myelin oligodendrocyte glycoprotein, CSF = cerebrospinal fluid.

**Figure 2:**
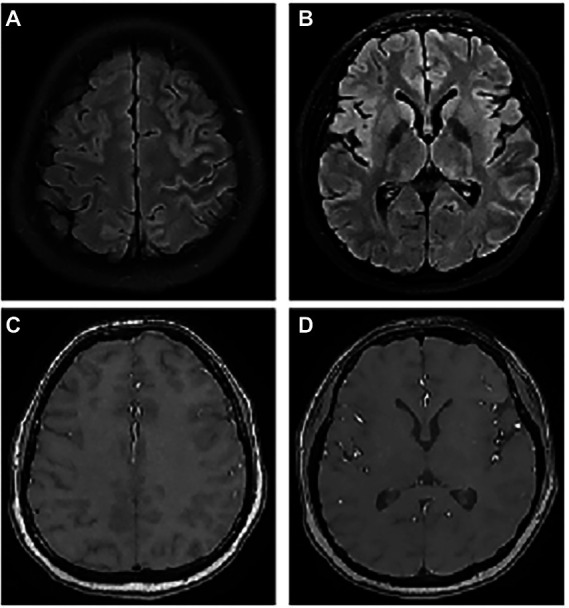
The abnormal MRI was obtained from patient 6. A 23-year-old woman with serum MOG antibodies present with headache only. Axial T2Flair images show hyperintense cortical signals in the left frontal, temporal, and parietal lobes **(A,B)**, but enhancement was normal **(C,D)**.

**Figure 3:**
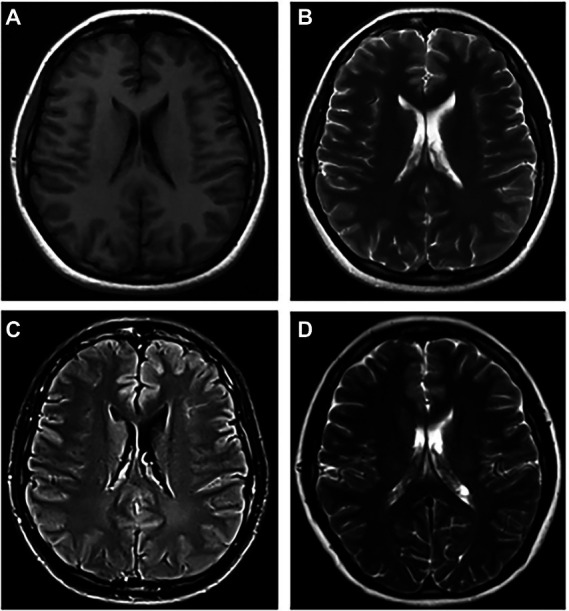
The abnormal MRI was obtained from patient 7. A 26-year-old woman with concomitant CSF MOG and NMDAR antibodies presenting on admission with severe headache, low-to-moderate fever, vision loss, and Limb jitter. Lumbar puncture show a significant increase in intracranial pressure, CSF pleocytosis and CSF protein elevation. Axial T1-weighted **(A)** and T2-weighted **(B)** images show normal in the left frontal lobe, but Flair image **(C)** appeared slight abnormal signal. After HIMP shock therapy, the patient leave the hospital with a decrease in laboratory indicators of CSF and no abnormalities on MRI **(D)**.

Among the nine patients, one patient had white matter involvement only, one had a deep gray matter involvement, and no abnormalities were found in one patient. All the remaining 6 had cortical involvement, with bilateral lesions in 2/6 (33.3%; Figure 1) andunilateral lesions in 4/6 (66.7%; Figures 2, 3). Except for 3 patients who did not receive enhanced MRI, 3/6 (50%) patients had meningeal or lesion enhancement. During the acute episode, five patients had white matter lesions, including 4/5 (80%) in the midline structures and 1/5 (20%) in the juxta-cortical white matter. All patients with MRI abnormalities showed low signal intensity on the T1 image and high signal intensity on the T2 image, which was more significant on the T2 flair phase.

### Treatment outcomes and recurrence

During the acute phase of the disease, eight patients (88.9%) received an intravenous high dose of methylprednisolone from the initial diagnosis, including a patient who received concurrent immunoglobulin therapy. One patient (11.1%) received a low-dose oral administration of prednisolone acetate (Table 1). Symptoms improved significantly in all patients. Seven patients (77.8%) recovered completely at discharge. Two patients (22.2%, Pat1, Pat7) had slightly different clinical symptoms, but they did not affect their normal life. Pat1 no longer had headaches, seizures, fever and weakness in the two legs, but his speech organization ability had not fully returned to normal, he could only complete brief verbal expressions. Pat7’s headache and fever disappeared and his vision returned to normal, but he was left with clinical symptoms of slight twitching of the bilateral upper limbs during fine motor performance.

All patients received a maintenance treatment with low-dose prednisolone acetate to prevent recurrence after discharge, and two patients (22.2%) presented a relapse during a median follow-up period of 9 months (range, 2–36 months), still meeting the diagnostic criteria for cortical encephalitis. One patient (Pat3) presented a relapse at the 10th month after discharge with serum MOG antibody (titer: 1:10) and was continuously positive for CSF MOG antibody (titer: 1:10), GFAP antibody (titer, 1:32) and NMDAR antibody (titer, 1:1). After the treatment with an intravenous high dose of methylprednisolone and additional mycophenolate mofetil, the patient totally recovered at discharge. Another patient (Pat8) had a recurrence at the third month after discharge, with clinical symptoms of lethargy, left upper limb paralysis, and blurred vision. Brain MRI showed left insula and gray matter with deep midline structures involvement, but MOG antibody was negative. Undetected MOG-Ab positive seroconversion or the pharmacological effect of prednisolone could not be excluded. The patient was treated again with prednisolone acetate tablets and achieved a significant clinical remission. The above evidence showed that the diagnosis of this patient was obtained even if the MOG antibody was negative.

## Discussion

This study described the clinical and imaging features of 9 adult patients with MOG antibody-associated cortical encephalitis, as well as the response to glucocorticoid therapy and recurrence. Our study revealed that adult patients with MOG antibody-associated cortical encephalitis frequently showed clinical manifestations similar to infections, such as headache, fever, seizure, CSF pleocytosis, intracranial hypertension and increased protein in CSF, manifestations that are different from the previously recognized clinical symptoms of adult patients with MOGAD (Sutton et al., 2022). However, no patient had an infection with pathogen. This result suggests that these non-specific symptoms might be a signal of MOG antibody-associated cortical encephalitis, thus deserving much attention to recognize the disease and avoid misdiagnosis. In addition, seizureis closely related to MOG antibody-associated encephalitis. Wang et al. reported that 50% of patients with MOG antibody-associated encephalitis develop seizures (Wang et al., 2019), and related studies suggested that generalized seizure is the main type of seizures in adults with MOG antibody-associated encephalitis (Ogawa et al., 2017; Zhong et al., 2019). Focal seizures are more common in children with MOG antigen-associated encephalitis (Armangue et al., 2020), suggesting that seizure types in MOG antibody-associated encephalitis might be age-related. In this study, 55.6% of adult patients also had seizures, and 80% presented generalized seizures, which was consistent with the conclusions of previous studies. Notably, two patients in our cohort (Pat7 and Pat9) had bilateral upper limb twitching, especially when holding objects or performing fine movements. This symptom was not reported up to now, and it might be related to the involvement of the motor cortex and its basal ganglia.

The MRI performance of MOG antibody-associated cortical encephalitis is distinctive, and it divides the lesions into unilateral lesions and bilateral medial frontal lesions, as reported by Fujimori et al. (2020b). Unilateral lesions are mostly located in the peripheral area of the middle cerebral artery, while bilateral lesions are mostly located in the peripheral area of the anterior cerebral artery, and the lesions are more visible on T2FLAIR image. Some patients also show the enhancement of the meninges or lesions (Yao et al., 2022). Our study also reached a similar conclusion, since all the eight patients with MRI abnormalities showed hyperintensity on T2 or Flair images. Among the six patients with cortical lesion, bilateral lesions were present in 2 of the 6 patients (33.3%), mainly distributed in the frontal and parietal cortex near the cerebral falx (Figure 1), while unilateral lesions were present in 4 of the 6 patients (66.7%), mainly distributed in the limbic cortex (Figures 2, 3). Moreover, patients with white matter lesions often showed abnormal signals in the midline structures (such as anterior commissure, tuber cinereum, foramen semiovale, corona radiata, and lateral ventricle) by MRI. In addition, the enrollment process of Pat2 and Pat4 in this study was troubled because they were initially diagnosed with ADEM. The MRI images of Pat2 only showed the involvement of bilateral thalamus. Although ADEM can also involve the thalamus, it is mostly with the concomitant lesion of the white matter (Pohl et al., 2016). The MRI images of the Pat4 patient showed only tiny unilateral white matter involvement, which was not consistent with the characteristics of large multifocal lesions of ADEM. Thus, these results were more consistent with the diagnostic criteria for encephalitis (Graus et al., 2016).

Regarding the pathogenicity of MOG antibodies in cortical encephalitis, some researchers describe entirely opposite points of view. Some scholars found no axonal and astrocyte damage and typical demyelination changes in the brain specimens of two patients with cortical encephalitis (Patterson et al., 2019), and CSF binding MBP, a marker of astrocyte damage, was not increased in patients with cortical encephalitis in the case of extensive cortical involvement and CSF pleocytosis (Ogawa et al., 2017). Hence, they speculate that MOG antibodies have little relevance in the occurrence of cortical encephalitis, and this disease may depend on some autoantibodies coexisting with MOG antibodies. Another group of researchers found that cortical demyelinating lesions are dominant as revealed by autopsy and brain biopsy of MOGAD patients, rather than white matter lesions compared with the pathological characteristics of patients with multiple sclerosis (Höftberger et al., 2020), which may suggest the potential pathogenicity of MOG antibodies.

In the present study, three patients had positive MOG antibody and NMDAR antibody in CSF (Pat4, Pat5, and Pat7), and clinical symptoms included headache, fever, seizures, and limb tremor. These symptoms overlap with those of anti-NMDAR encephalitis, as well as the imaging findings, although they did not show other typical symptoms such as mental and behavioral abnormalities, memory loss and speech disorder (Dalmau et al., 2011), which could be related to the early immunotherapy. Moreover, Ding et al. reported that approximately 9% of serum MOG-Ig G+ patients also had CSF anti-NMDAR-Ig G+, and no difference in clinical symptoms was found between anti-NMDAR-IgG+/MOG-IgG+ patients and anti-NMDAR-IgG+/MOG-IgG− patients (Ding et al., 2021). Besides, a previous study showed that oligodendrocytes express NMDAR. Therefore, it is possible that NMDAR is simultaneously involved when the immune attack against myelin sheath occurs (Zhou et al., 2017). Then, the presence of anti-NMDAR antibody reduces the expression of cell surface receptors, eventually leading to neuronal dysfunction (van Coevorden-Hameete et al., 2014). This further confirms that anti-NMDAR antibody might be pathogenic. In addition, one patient had positive MOG antibody, NMDAR antibody and GFAP antibody in CSF (Pat3). Meningoencephalitis and subcortical white matter lesions are the main manifestations of GFAP astrocytopathy, while seizures and cortical lesions are rare (Shan et al., 2018). Therefore, GFAP antibody also may not play a dominant role in the course of the disease. Taken together, these pieces of evidence, together with our results revealed the need for subsequent studies to determine whether anti-MOG antibodies are merely “concomitant antibodies” in cortical encephalitis. However, the response to the treatment revealed that MOG antibody-associated cortical encephalitis was sensitive to glucocorticoids (Ogawa et al., 2017; Tian et al., 2021; Yao et al., 2022), which is consistent with the response to the treatment and clinical prognosis of MOGAD (Ambrosius et al., 2020), but markedly different from anti NMDAR encephalitis. Titulaer and Dalmau et al. found that nearly half of anti-NMDAR encephalitis patients did not obtain an effective relief of symptoms after receiving first-line immunotherapy (steroid hormone, IVIG and plasma exchange alone or in combination), and second-line immunotherapy (including rituximab andcyclophosphamide) was usually needed (Dalmau et al., 2011; Titulaer et al., 2013). Therefore, it is reasonable to believe that MOG antibodies act as the “responsible antibodies” of cortical encephalitis.

An intravenous high-dose of methylprednisolone has become the most common treatment to combat MOGAD in the acute phase, but there is no consensus on the necessity and drugs of long-term immunotherapy for MOGAD patients (Klein da Costa et al., 2021). In our study, the clinical symptoms of eight patients receiving a high dose of methylprednisolone were alleviated or disappeared. Nine patients received low-dose oral glucocorticoid as a long-term immunotherapy. Only two patients showed a relapse after a median follow-up of 9 months. After receiving the same treatment after the last attack, the clinical symptoms were improved, suggesting that the treatment with glucocorticoids or long-term immunotherapy might be a good choice for MOG antibody-related cortical encephalitis.

Previous studies found that MOG antibody titer in MOGAD patients was higher in the acute phase than in the remission, and patients with high antibody titer and consistently positive antibodies were more likely to have a recurrent course of disease (Di Pauli et al., 2011; Jarius et al., 2016; Cobo-Calvo et al., 2018; Reindl and Waters, 2019). Although MOG antibody associated cortical encephalitis is considered as a type of MOGAD, the research of Wang et al. shows that MOG antibody titers vary widely in patients with cortical encephalitis and are independent of disease severity and prognosis (Wang et al., 2021). The present study reached the same conclusion, but this result might be explained by the limited sample size. Therefore, multi-center, large-sample studies are needed to explore the relationship between MOG antibody titers and the severity and prognosis of cortical encephalitis.

Despite the promising results, our research also has several limitations. First, our sample size was too small and too narrow, only involving a hospital affiliated to a medical university. Secondly, the clinical data of patients are insufficient and the manuscript lacks direct comparisons with outcomes of MOGAD without encephalitis. Finally, the short follow-up and limited sample size might lead to an overestimation of the efficacy of glucocorticoids and underestimation of the recurrence rate of the disease.

## Conclusion

The clinical manifestations of adult MOG antibody associated-cortical encephalitis are significantly different from those of typical MOGAD. In the acute phase of the disease, patients are prone to show signs similar to CNS infection, requiring clinicians to have the ability to recognize the disease to avoid misdiagnosis. In addition, seizures are common in MOG antibody-related encephalitis, and the type of seizures is age-related. Brain MRI results showed that the distribution of cerebral cortex lesions is closely related to the classification of encephalitis. Based on the patient’s response to the treatment, glucocorticoids might be effective to combat MOG antibody-associated cortical encephalitis, which is consistent with the treatment response and clinical prognosis of MOGAD. Therefore, our opinion was that MOG antibody might be the responsible antibody in MOG antibody-associated cortical encephalitis, although further studies are needed to confirm this hypothesis.

## Data availability statement

The original contributions presented in the study are included in the article/supplementary material, further inquiries can be directed to the corresponding author.

## Ethics statement

The studies involving human participants were reviewed and approved by The Ethics Committee of the Affiliated Brain Hospital of Nanjing Medical University. Written informed consent to participate in this study was provided by the participants’ legal guardian/next of kin.

## Author contributions

YW drafted the manuscript, designed the study, and analyzed the data. HZ analyzed the data again and revised the manuscript. XC and LL contributed to the acquisition of data. DZ participated in the data analysis. JL contributed to patients and data recruiting, revised the manuscript and provided financial support. All authors contributed to the article and approved the submitted version.

## Funding

The study is financially sponsored by the National Natural Science Research Foundation of China (81500969).

## Conflict of interest

The authors declare that the study was conducted in the absence of any commercial or financial relationships that could be construed as a potential conflict of interest.

## Publisher’s note

All claims expressed in this article are solely those of the authors and do not necessarily represent those of their affiliated organizations, or those of the publisher, the editors and the reviewers. Any product that may be evaluated in this article, or claim that may be made by its manufacturer, is not guaranteed or endorsed by the publisher.
